# Trajectory and predictive factors of cancer-related fatigue in hospitalized elderly patients with non-small cell lung cancer undergoing chemotherapy

**DOI:** 10.1097/MD.0000000000045277

**Published:** 2026-02-13

**Authors:** Chen Liu, Hao Wang, Jiaxuan Wen, Taotao Xu, Han Wang, Xiuhong Wei, Yan Zhai

**Affiliations:** a School of Nursing, Weifang Medical University, Weifang, P.R. China; b Department of Oncology, Weifang People’s Hospital, Weifang, P.R. China; c Department of Dermatology, Weifang People’s Hospital, Weifang, P.R. China; d School of Nursing, Shandong Xiandai University, Weifang, P.R. China; e Department of Health Management Center, Weifang People’s Hospital, Weifang, P.R. China.

**Keywords:** cancer-related fatigue, elderly, latent class growth model, non-small cell lung cancer, predictive factors

## Abstract

This study examines the demographic and clinical characteristics of elderly non-small cell lung cancer patients undergoing chemotherapy, focusing on levels of cancer-related fatigue (CRF), anxiety, depression, sleep, and social support. The goal is to explore the trajectory and predictive factors of CRF development to aid in patient coping. Conducted from October 2022 to October 2023 in a tertiary hospital in Weifang, the longitudinal study assessed CRF, anxiety, depression, sleep quality, and social support at 4 time points during chemotherapy. Repeated measures ANOVA, *t*-tests, one-way ANOVA, and Pearson correlation analysis were used to evaluate data. Mplus 8.3 software established a latent class growth model (LCGM) to track CRF trajectories, and multinomial logistic regression identified predictive factors for CRF classes. Cancer-related fatigue scores: mean CRF scores at T1, T2, T3, and T4 were 19.33, 28.40, 32.06, and 26.12, respectively. CRF peaked at T3 and then gradually declined, with significant differences in CRF, anxiety, depression, sleep quality, and social support across T1-T4 (*P* < .05). Significant factors affecting CRF included disease stage, treatment regimen, recurrence, anxiety and depression levels, sleep quality, and social support. Three CRF trajectory classes were identified with the best data fit: low-level slow increase (20 patients), high-level gradual relief (35 patients), and low-level rapid increase (53 patients). Predictors for the high-level gradual relief group included disease stage, anxiety score, and sleep quality score (*P* < .05). For the low-level rapid increase group, predictors were disease stage, anxiety score, sleep quality score, and social support (*P* < .05). CRF in elderly non-small cell lung cancer patients undergoing chemotherapy is influenced by disease characteristics, psychological status, sleep quality, and social support. Three distinct CRF trajectories were identified, with disease stage, anxiety, depression, and sleep quality serving as key predictive factors.

## 1. Introduction

Lung cancer is one of the leading causes of cancer-related deaths worldwide, with high incidence and mortality rates. According to the 2020 World Cancer Statistics, approximately 2.2 million new cases of lung cancer are diagnosed annually, resulting in about 1.8 million deaths. This accounts for 11.4% of all diagnosed cancers and 18.0% of all cancer deaths. Among women, lung cancer is the third most common cancer, following breast cancer and colorectal cancer.^[1]^ According to the World Health Organization, lung cancer is the leading cause of cancer deaths globally, with the highest mortality rates among both men and women.^[2]^ Especially in China, lung cancer has the highest incidence and mortality rates among all cancers, accounting for 18.06% of new cases and 20.9% of cancer deaths.^[3]^ Lung cancer is broadly classified into 2 main types: non-small cell lung cancer (NSCLC) and small cell lung cancer (SCLC), with SCLC accounting for 15% to 20% of all lung cancer cases.^[4]^

In its latest guidelines, the National Comprehensive Cancer Network defines high-risk individuals as those aged 50 and older with a smoking history of at least 20 pack-years (calculated as the number of packs smoked per day multiplied by the number of years smoked). As people age, their bodily functions decline and their immune system becomes less efficient at eliminating cancer cells. Consequently, individuals over the age of 65 have an 11-fold higher cancer incidence compared to those under 65.^[5]^ An analysis of nearly 40 years of case data in the United States shows that the proportion of elderly lung cancer patients is increasing.^[6]^ With the increasing aging population in our country, the incidence of elderly NSCLC continues to rise.^[7]^ The latest statistics show that currently, over 50% of lung cancer cases occur in individuals aged 70 and above, with 14% of patients being over 80 years old.^[8]^ As the most common type of lung cancer, NSCLC affects over 70% of patients aged 65 and older, with those over 75 constituting 37% of cases. Furthermore, over 50% of patients are diagnosed at an advanced stage.^[9,10]^ NSCLC often presents without noticeable clinical symptoms in its early stages, leading to most patients being diagnosed in advanced stages. For elderly patients, by the time it’s detected, surgical treatment opportunities are often lost. For those ineligible for surgery, medical teams typically employ curative synchronous or sequential radiotherapy and chemotherapy regimens to prolong survival and enhance quality of life as much as possible.^[3]^ With the exploration of various new drugs, technologies, and treatment modalities, the survival of elderly NSCLC patients has been extended, alleviating physical suffering. However, they also endure various degrees of adverse reactions. Chemotherapy drugs are associated with severe gastrointestinal reactions,^[11]^ leading to hair loss.^[12]^ cancer-related fatigue (CRF), shortness of breath,^[13]^ and cognitive impairments.^[14]^ The diagnosis and adverse reactions from chemotherapy can significantly impact the psychological well-being of patients and their families. During treatment, NSCLC patients often experience anxiety and guilt about the progression of the disease, treatment costs, and the burden on their families.^[15]^ NSCLC imposes significant psychological and physiological stress on patients, while also consuming substantial healthcare resources and increasing societal burdens.

Among many adverse reactions, CRF occurs frequently and persists for extended periods.^[16–18]^ It is also considered one of the most distressing adverse reactions in cancer treatment.^[19]^ CRF is a complex condition influenced by multiple dimensions, including physical, emotional, cognitive, psychological, social, and spiritual factors.^[20]^ Unlike fatigue from typical activities, CRF is severe pathological fatigue caused by disease and treatment factors. It manifests more intensely, aggressively, and persistently, making it difficult to alleviate through rest. Due to variations in patient populations, treatment methods, and assessment approaches, estimates of CRF prevalence range widely from 25% to 99%. Among lung cancer patients specifically, the occurrence rate of CRF reaches as high as 98%.^[21]^ During radiation therapy, chemotherapy, hormone therapy, or biological therapy, 30% to 60% of patients report worsening fatigue, reaching moderate to severe levels. In some cases, this fatigue may directly lead to treatment discontinuation. Additionally, 25% to 30% of patients continue to experience fatigue for years after treatment completion.^[22]^ CRF overlaps with comorbidities in lung cancer survivors and significantly interferes with Health-related Quality of Life (HRQoL). Patients emphasize that CRF severely impacts their lives, necessitating reliance on others, compromising decision-making abilities, and hindering normal daily functioning. It also has a notable impact on professional and social relationships.^[23]^ Moreover, severe CRF can also shorten patients’ overall survival.^[24]^ In recent years, researchers have focused on identifying the mechanisms of CRF, proposing various hypotheses. The specific mechanisms of CRF are highly complex and currently contentious. Several hypotheses exist, such as cytokine imbalance, central neurotransmitter dysregulation, serotonin dysregulation, and dysfunction of the hypothalamic-pituitary-adrenal axis. Cytokines have received particular attention. CRF in patients receiving anthracycline chemotherapy has been linked to interleukin-6 (IL-6), interleukin-1 receptor antagonist (IL-1RA), transforming growth factor β (TGF-β), and soluble tumor necrosis factor receptor 2 (sTNF-R2).^[24]^ Radiation therapy can release cytokines from tissues, and levels of cytokines during radiation therapy are positively correlated with fatigue levels. Elevated levels of cytokines such as IL-1β, IL-2, IL-6, and TNF-α are significantly associated with the severity of fatigue.^[25]^ It is evident that radiation therapy is also directly associated with CRF.

The factors influencing CRF are complex, and numerous studies have explored its determinants. Current research analyzes the impact of general demographics, psychological health status, clinical characteristics, nutritional status, and other factors on CRF. Knobel et al found that in lymphoma patients, CRF is gender-related, with females experiencing higher levels of fatigue.^[26]^ Research by Hongbo et al shows that among lung cancer patients, older age is associated with more severe CRF.^[27]^ Davis et al found that chemotherapy patients experience disrupted sleep quality, and prolonged sleep deprivation exacerbates fatigue symptoms, making it more difficult for patients to alleviate fatigue.^[28]^ Long et al’s study suggests that respiratory difficulties have a higher direct impact on fatigue among lung cancer patients. They recommend prioritizing the management of respiratory difficulties over coughing when addressing fatigue.^[29]^ Research by Poort et al indicates that CRF is closely associated with patients” psychological health. Patients with higher levels of depression are more prone to fatigue.^[30]^ Additionally, research by Huang et al suggests that the more advanced the clinical stage of the tumor, the poorer the physical functioning, and consequently, the higher the risk of fatigue among cancer patients.^[31]^ Although existing research has explored numerous factors influencing CRF, it lacks guidance from theoretical frameworks and often takes a narrow analytical perspective. Currently, there is limited research in China on the evolving trends of CRF and further enrichment is needed in studying related predictive factors. The state-of-the-art table showing the research gap is presented in Table 1.

**Table 1:** State-of-the-art summary highlighting the research gap.

Study/author	Focus	Findings	Research gap
Knobel et al^[26]^	Gender-related fatigue	Female lymphoma patients experience higher levels of cancer-related fatigue.	This study is narrow, only proving the relationship between gender and fatigue.
Hongbo et al^[27]^	Age-related fatigue	Older lung cancer patients experience more severe cancer-related fatigue.	The sample size in this study is small, and it lacks rigorous statistical analysis.
Mellar et al^[28]^	Sleep disruption and fatigue	Chemotherapy patients with disrupted sleep quality experience exacerbated fatigue.	Lack of a validated tool to measure fatigue.
Hoang Long et al^[29]^	Respiratory difficulties and fatigue	Respiratory difficulties have a higher direct impact on cancer-related fatigue among lung cancer patients.	This study used a convenience sampling method and was designed as a cross-sectional study
Hanneke et al^[30]^	Psychological health and fatigue	Higher levels of depression are associated with more cancer-related fatigue.	Only examined patients without persistent or recurrent disease
Huang et al^[31]^	Clinical stage and physical function impact	Advanced tumor stages and poorer physical functioning are linked to higher cancer-related fatigue risk.	low evidence-based strength.

This study referenced the theory of Symptoms Experience in Time.^[32]^ In 2003, Henly and colleagues introduced the Symptoms Experience in Time theory.^[33]^ The theory integrates and expands upon traditional symptom experience theories such as the Theory of Unpleasant Symptoms, the Theory of Symptom Management, and the Chemotherapeutic Intervention for postsurgical Pain. These existing theories all to some extent involve the dimension of time, whereas SET theory explicitly recognizes time as a core element in the experience and management of symptoms. In theory, symptom experience is seen as a continuous process, where time is divided into 4 main dimensions: clock time, biological and social time, perceptual time, and transcendent time. Each dimension profoundly influences symptom experience and management. SET theory likens the evolution of symptoms to the operation of a computer program, encompassing stages like input, decision points, operations, and outputs. Additionally, this theory extends the impact factors of traditional symptom theory by integrating fundamental nursing concepts – person, environment, and health. Symptom assessment involves perception, time, distress, severity, and nature (PTDIQ). The SET theory emphasizes the importance of incorporating time dimensions into symptom research and advocates for longitudinal studies to deepen understanding of time-dependent phenomena in symptom experience and management. By providing a comprehensive theoretical framework for symptom management, SET aids in designing and validating effective symptom management strategies and evaluating treatment outcomes. Guided by SET, this study considers CRF in chemotherapy patients as a dynamic process over time. Following diagnosis, patients undergo multiple cycles of chemotherapy, during which CRF exhibits dynamic changes influenced by environmental factors, time progression, and treatment stages. This understanding builds upon previous research and chemotherapy cycles in elderly NSCLC patients.^[34,35]^ This study conducted longitudinal surveys with patients at 4 different time points: after determining the chemotherapy regimen (T1), after the first completion of chemotherapy (T2), after the fourth completion of chemotherapy (T3), and after the completion of chemotherapy (T4).

## 2. Materials and methods

### 2.1. Study subjects

#### 2.1.1. Selection of study subjects

Patients with NSCLC undergoing chemotherapy in the oncology department of a tertiary hospital in Weifang City from April 2023 to October 2023 were selected as study subjects.

Inclusion criteria: Patients diagnosed with NSCLC confirmed by histopathology; patients who have chosen a chemotherapy regimen, are receiving standard cycle chemotherapy, and are expected to complete the treatment cycle; patients aged 60 years or older; patients who have provided informed consent and are capable of answering clearly.

Exclusion criteria: Patients who have difficulty understanding the questionnaire items; patients with other serious complications or concurrent malignancies.

Exclusion or dropout criteria: Patients who discontinue treatment within the treatment cycle due to intolerance to the chemotherapy regimen; patients for whom the survey cannot continue due to transfer to another hospital or death.

#### 2.1.2. Estimation of sample size

The sample size was estimated using PASS 11.0 software (NCSS, Kaysville), following methods for longitudinal study sampling surveys. A confidence level of 0.95, significance level (α) of 0.05, and allowable error of 0.1 were specified. Based on the calculation formula $n=\frac{z_{\alpha /2\;}p\left(1-p\right)}{\delta^{2}}$ and accounting for potential sample loss during the survey, assuming a sample recovery rate (p) between 70% and 90%., the final estimated sample size is approximately 60 to 140 cases.

### 2.2. Research methods

This is a longitudinal survey study, conducting baseline assessments with patients after definitive diagnosis, and evaluations at the following time points: after determining the chemotherapy regimen (T1), after the first completion of chemotherapy (T2), after the fourth completion of chemotherapy (T3), and after the completion of chemotherapy (T4).

### 2.3. Research tools

#### 2.3.1. General and disease-related data collection form

This study employs a self-designed questionnaire to collect demographic and medical characteristics of patients. Demographic characteristics include: age, gender, body mass index (BMI), education level, marital status, income level, and medical insurance status. Medical characteristics include: cancer histopathological type, cancer staging, treatment regimen, and recurrence status. Medical information is sourced from medical records, attending physicians treating the patients, or head nurses in the patient’s ward.

#### 2.3.2. Cancer fatigue scale (CFS)

The cancer fatigue scale (CFS) was designed by Okuyama in the year 2000.^[36]^ CFS consists of 3 dimensions and 15 items in total. These dimensions are the physical component scale, the emotional component scale, and the cognitive component scale. The CFS demonstrates good stability (test–retest reliability of 0.69, *P* < .001) and strong internal consistency (Cronbach α coefficient of 0.88 across the 15 items). Each item on the scale is scored from 0 to 4, with higher scores indicating greater levels of CRF. The total score ranges from 0 to 60, with the physical component scale accounting for 28 points, and the emotional and cognitive component scales each accounting for 16 points. Due to its simplicity and ease of understanding, the CFS is widely used. The Chinese version of the CFS was translated and validated by Fengling et al in 2011.^[37]^ The scale has been validated with Cronbach α coefficients ranging from 0.63 to 0.86 for internal consistency and test–retest reliability between 0.55 and 0.77. Currently, the Chinese version of the CFS is widely used for assessing CRF domestically and is relatively mature. Based on scoring, fatigue levels are categorized into 4 grades: scores of 0 to 5 indicate no CRF, 6 to 15 indicate mild CRF, 16 to 30 indicate moderate CRF, and 31 to 60 indicate severe CRF.

#### 2.3.3. Assessment scale for anxiety, depression, sleep quality, and social support

The hospital anxiety and depression scale (HADS) is one of the most commonly used questionnaires for screening anxiety and depression in hospital settings. It was developed by Zigmond et al in 1983.^[38]^ Annunziata et al demonstrated that HADS is effective in identifying anxiety and depression in cancer patients, making it suitable for clinical practice.^[39]^ HADS shows good internal consistency (Cronbach α coefficients for total score, anxiety subscale, and depression subscale are 0.879, 0.806, and 0.806 respectively), with item test–retest reliabilities ranging from 0.871 to 0.988.^[40]^ The Athens Insomnia Scale (AIS) was developed by Soldatos et al in 2010 based on the diagnostic criteria from the International Classification of Diseases.^[41,42]^ The scale has a Cronbach α coefficient of 0.820 and a test–retest reliability of 0.840. The social support rating scale was designed by Shuiyuan in 1986 and includes 10 items across 3 dimensions: active support, objective support, and the utilization of support.^[43]^ This scale is widely used in social support research and has demonstrated good reliability and validity (Cronbach α ranges from 0.825 to 0.896, and test–retest reliability is 0.92).^[44]^

### 2.4. Data collection

Data collection relies on questionnaires and medical record reviews. Personnel involved in data collection receive training to explain important considerations for the survey, ensuring objective and accurate collection without using leading language and maintaining a rigorous approach. The survey is conducted face-to-face by the interviewer, who guides or assists patients in filling out the questionnaire. The respondents complete all questionnaires face-to-face at the following time points: after determining the chemotherapy regimen (T1), after the first completion of chemotherapy (T2), after the fourth completion of chemotherapy (T3), and after the completion of chemotherapy (T4). The collected questionnaires are immediately checked for errors or omissions to ensure their authenticity, completeness, and validity. After collection and verification, the researchers and interviewers use EpiData 3.1 to enter the data into the computer for backup. Depending on the type of data, appropriate statistical methods are selected for analysis, and statistical experts are consulted when necessary to ensure reliable data analysis.

### 2.5. Ethical principles

This study obtained prior approval and was registered with relevant hospital departments. Investigators explained the purpose and significance of the study to patients to ensure their informed consent and pledged to protect patient privacy. The entire survey process adheres to ethical principles of informed consent, respect for individuals, equality, and non-harm.

### 2.6. Statistical methods

SPSS 26.0 (Chicago) and Mplus 8.3 (Muthén & Muthén, Los Angeles) were used for data processing. A *P*-value of < .05 indicates statistical significance. Count data from general and disease-related information are expressed as frequency (n) and percentage (%), while measurement data are expressed as mean ± standard deviation. The Kolmogorov–Smirnov test (K-S test) was used to test the normality of the data. Data from the 4 measurements that conform to a normal distribution were analyzed using repeated measures analysis of variance (ANOVA), and variables that do not conform to a normal distribution were analyzed using nonparametric tests to examine differences in CRF, anxiety, depression, sleep quality, and social support scores. Independent sample *t*-tests or one-way ANOVA were used to test for differences in fatigue scores and general and disease-related information across the 4 surveys. Pearson correlation analysis or Spearman correlation analysis was used to explore the relationship between anxiety and depression levels, sleep quality, social support, and CRF in patients. Pearson correlation analysis was used for normally distributed data, and Spearman correlation analysis was used for non-normally distributed data. Mplus 8.3 software was used to establish a latent class growth model (LCGM) to fit the developmental trajectory of CRF in elderly NSCLC chemotherapy patients. The raw CRF values at 5 time points were used as the data for model fitting. Parameters were estimated using the robust maximum likelihood estimator, and model fit was evaluated using the following indicators: loglikelihood (LL), information criteria including Akaike information criterion (AIC), Bayesian information criterion (BIC), and sample size-adjusted BIC (aBIC), with smaller values indicating better model fit, and aBIC being the most optimal; Entropy, which ranges from 0 to 1, is used to assess the precision of trajectory classification, with values above 0.80 indicating a precise trajectory model. Model comparisons were conducted using the bootstrapped likelihood ratio test (BLRT) and Vuong-Lo-Mendell-Rubin likelihood ratio test (VLMR), comparing the current model with *k* classes to the model with *k* − 1 classes, with *P*-values < .05 indicating that the model with k trajectories fits better. Multinomial logistic regression analysis was used to determine the predictive effects of general and disease-related information, as well as patient anxiety and depression levels, sleep quality, and social support, on fatigue trajectory categories.

## 3. Results

### 3.1. General demographic and medical characteristics

This study included 120 NSCLC patients. Twelve patients discontinued, leaving 108 with complete data, resulting in an effective questionnaire rate of 89.2%. There were 60 male patients (55.6%), with 62 patients aged 60 to 70 years (57.4%), and an average age of 70.3 years. Over 50% were overweight or obese, 16.7% were elderly without a spouse, 69.3% had incomes above 3000, and 97% had health insurance. The majority had adenocarcinomas (48.1%), 62.1% were in stages III/IV, with few surgical opportunities, and 69.5% underwent chemoradiotherapy. Over 80% were newly diagnosed with NSCLC. Specific details are provided in Table 2.

**Table 2:** General demographic and medical characteristics (n = 108).

Item	Category	Number (n)	Percentage (%)
Gender	Male	60	55.6
	Female	48	44.4
Age	60 ≤ age < 70	62	57.4
	70 ≤ age < 80	38	35.2
	≥ 80 yr	8	7.4
BMI	BMI < 18.5	39	36.1
	18.5 ≤ BMI < 24	15	13.9
	BMI ≥ 24	54	50
Marital status	Married	90	83.3
	Single/divorced/widowed	18	16.7
Monthly family income	<3000	33	30.6
	3000 ≤ Income < 5000	54	50
	≥5000	21	19.4
Insurance status	Insured	105	97.2
	Self-paying	3	2.8
Disease type	Squamous cell carcinoma	33	30.6
	Adenocarcinoma	52	48.1
	Large cell carcinoma	8	7.4
	Others	15	13.9
Disease stage	Stage I/II	41	37.9
	Stage III/IV	67	62.1
Treatment regimen	Chemoradiotherapy	75	69.5
	Chemotherapy	25	23.1
	Surgery + Chemoradiotherapy	8	7.4
Recurrence	Initial	89	82.4
	Recurrent	19	17.6

BMI = body mass index.

### 3.2. Scores and trends of CRF and related factors in elderly NSCLC patients

#### 3.2.1. CRF scores and trends in elderly NSCLC patients from T1 to T4

**Figure 1.**
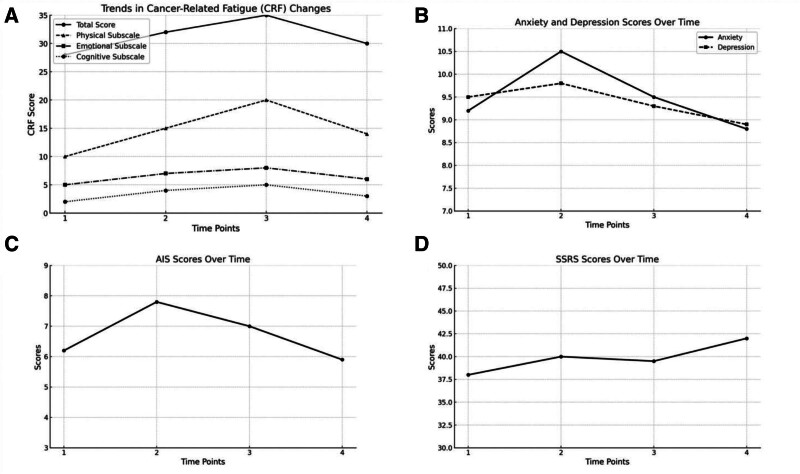
(A) cancer-related fatigue trends in elderly non-small cell lung cancer patients from T1 to T4. (B) HADS scores and trends in elderly non-small cell lung cancer patients. (C) AIS scores and trends in elderly non-small cell lung cancer patients. (D) Social support scores and trends in elderly non-small cell lung cancer patients. AIC = Akaike information criterion, HADS = hospital anxiety and depression scale.

The fatigue levels of the patients gradually increased, peaking at T3 and then decreasing at T4, as shown in Figure 1. Even after the treatment cycle ended, the fatigue levels remained higher than before the treatment, with statistically significant differences in CFS scores between T1 and T4 (*P* < .05). Specifically, physical fatigue also showed a trend of first rising and then falling, emotional fatigue gradually decreased, and cognitive fatigue increased as the treatment progressed, as seen in Table 3 and Figure 1A.

**Table 3:** Current status of cancer-related fatigue and related factors during different chemotherapy periods (n = 108, $\bar{x}\pm \;s$).

Dimension	Score				Time effect		Between-group effect		Interaction effect		Pairwise comparison
	T1	T2	T3	T4	*F*	*P*	*F*	*P*	*F*	*P*	
Total score of the scale	19.33 ± 3.65	28.40 ± 5.23	32.06 ± 4.37	26.12 ± 4.12	245.65	<.001	153.25	<.001	106.35	.007	*,†,‡,§,‖,¶
Physical subscale	7.72 ± 2.87	13.98 ± 4.29	19.06 ± 3.28	13.18 ± 3.17	263.14	.017	192.71	.021	127.63	.039	*,†,‡,§,¶
Emotional subscale	8.17 ± 2.05	9.12 ± 2.10	7.48 ± 2.20	7.06 ± 2.14	40.76	<.001	19.83	.004	11.24	.015	*,‡,§,‖
Cognitive subscale	3.44 ± 1.14	5.30 ± 1.67	5.52 ± 1.74	5.89 ± 1.95	69.53	<.001	47.224	.012	24.52	.036	*,†,‡,§
Anxiety subscale	9.10 ± 2.34	9.97 ± 2.73	9.09 ± 2.95	8.66 ± 2.45	7.536	<.001	4.801	.003	2.014	.017	‖
Depression subscale	9.95 ± 2.71	9.49 ± 2.87	8.94 ± 2.52	8.30 ± 2.29	15.245	<.001	8.142	<.001	4.574	<.001	†,‡,¶
AIS score	6.01 ± 3.93	7.95 ± 4.92	7.38 ± 4.79	5.54 ± 4.06	11.579	<.001	6.555	<.001	3.547	<.001	*,‖,¶
SSRS score	37.78 ± 14.45	40.32 ± 13.69	40.67 ± 13.83	43.37 ± 14.50	5.453	.009	2.808	.040	1.038	.081	‡

AIS = Athens Insomnia Scale, SSRS = social support rating scale.

* Significant difference between T1 and T2.

† Significant difference between T1 and T3.

‡ Significant difference between T1 and T4.

§ Significant difference between T2 and T3.

‖ Significant difference between T2 and T4.

¶ Significant difference between T3 and T4.

#### 3.2.2. HADS scores and trends in elderly NSCLC patients from T1 to T4

The patients’ anxiety levels increased at T2, but the difference was not statistically significant. Depression levels gradually decreased as chemotherapy progressed. as seen in Table 3 and Figure 1B.

#### 3.2.3. AIS scores and trends in elderly NSCLC patients from T1 to T4

Patients experienced the worst sleep quality at T2, which improved during subsequent treatment phases, showing significant improvement at T4. However, the difference compared to T1 was not statistically significant. The AIS scores and the proportion of patients experiencing insomnia are presented in Table 3 and Figure 1C.

#### 3.2.4. Social support scores and trends in elderly NSCLC patients from T1 to T4

There was a statistically significant difference in social support scores between T1 and T4 (*P* = .040), indicating an improvement in the patients’ social support status during the treatment process. The social support scores and the specific proportions of social support from T1 to T4 are shown in Table 3. The trend of social support score changes from T1 to T4 is depicted in Figure 1D.

### 3.3. Impact factors analysis of CRF in elderly NSCLC patients

#### 3.3.1. Correlation between CRF and general and disease-related conditions in elderly NSCLC patients

In general demographics, including age, gender, BMI, education level, marital status, income level, and medical insurance status, there were no statistically significant differences in CRF across the 4 time points. Among medical characteristics, differences in cancer pathology subtypes showed no statistical significance regarding CRF. However, disease analysis and treatment regimens significantly influenced CRF levels across all 4 time points, showing statistically significant differences as depicted in Table 4.

**Table 4:** Univariate analysis of cancer-related fatigue levels from T1 to T4 (n = 108, $\bar{x}\pm \;s$).

Item	T1	T2	T3	T4
Gender				
Male	19.73 ± 3.69	28.63 ± 5.18	31.65 ± 4.40	26.23 ± 4.02
Female	18.83 ± 3.62	28.10 ± 5.39	32.58 ± 4.37	25.98 ± 4.33
*t*	1.27	-0.55	-1.10	0.32
*P*	.89	.79	.96	.70
Age				
60 ≤ age < 70	19.19 ± 3.67	28.15 ± 5.51	32.25 ± 4.11	26.07 ± 4.02
70 ≤ age < 80	19.88 ± 3.38	28.59 ± 4.84	32.38 ± 4.99	26.00 ± 4.43
≥ 80 yr	18.83 ± 4.88	29.67 ± 5.65	28.67 ± 3.01	26.67 ± 4.50
*F*	0.41	0.51	2.58	0.25
*P*	.89	.6	.08	.78
BMI				
BMI < 18.5	26.16 ± 4.33	28.50 ± 5.45	31.63 ± 4.56	25.96 ± 4.25
18.5 ≤ BMI < 24	26.84 ± 4.34	28.95 ± 4.95	31.68 ± 3.65	26.42 ± 3.75
BMI ≥ 24	25.83 ± 3.98	27.97 ± 5.24	32.86 ± 4.50	26.19 ± 4.28
*F*	0.42	0.23	0.93	0.09
*P*	.66	.79	.40	.91
Marital status				
Married	19.40 ± 3.61	28.34 ± 5.32	31.91 ± 4.41	26.29 ± 4.05
Unmarried/divorced/widowed	19.00 ± 3.23	28.67 ± 5.24	32.83 ± 4.50	25.28 ± 4.20
*t*	0.42	-0.24	-0.81	0.95
*P*	.37	.83	.63	.44
Monthly family income				
<3000	19.76 ± 3.87	28.79 ± 5.60	32.44 ± 4.55	26.21 ± 4.63
3000 ≤ income < 5000	18.78 ± 3.58	28.28 ± 5.21	32.48 ± 4.31	26.13 ± 3.91
≥5000	20.1 ± 3.49	28.05 ± 5.01	30.30 ± 4.12	25.95 ± 4.07
*F*	1.30	0.15	2.02	0.02
*P*	.28	.86	.14	.98
Insurance status				
Insured	19.32 ± 3.71	28.44 ± 5.26	32.22 ± 4.33	26.15 ± 4.17
Self-paying	19.67 ± 2.08	27.00 ± 6.00	26.67 ± 3.21	25.00 ± 3.61
*t*	-0.16	0.47	2.20	0.47
*P*	.19	.81	.48	.54
Disease type				
Squamous cell carcinoma	18.82 ± 3.83	28.56 ± 5.29	32.18 ± 4.14	26.91 ± 4.12
Adenocarcinoma	19.62 ± 3.62	28.50 ± 5.45	31.63 ± 4.56	25.96 ± 4.25
Large cell carcinoma	19.67 ± 4.30	28.56 ± 5.41	31.00 ± 4.61	25.44 ± 3.32
Other	19.31 ± 3.22	27.46 ± 4.75	34.23 ± 3.94	25.15 ± 4.36
*F*	0.34	0.15	1.42	0.75
*P*	.80	.93	.24	.53
Disease stage				
Stage I/II	14.63 ± 2.13	20.88 ± 2.73	25.75 ± 2.24	27.01 ± 3.66
Stage III/IV	20.21 ± 3.24	29.78 ± 4.42	33.18 ± 3.69	21.00 ± 2.85
*t*	6.57	7.72	7.77	6.23
*P*	.03	<.01	.02	.01
Treatment regimen				
Chemotherapy	16.88 ± 3.92	24.79 ± 5.04	29.17 ± 4.36	24.67 ± 4.09
Chemoradiotherapy	19.92 ± 3.02	29.09 ± 4.59	32.76 ± 4.15	26.23 ± 4.13
Surgery + chemoradiotherapy	21.00 ± 4.74	32.22 ± 5.36	34.00 ± 4.47	29.11 ± 3.95
*F*	8.27	10.18	7.95	4.07
*P*	<.00	<.00	<.01	.02
Recurrence status				
First occurrence	23.10 ± 2.86	34.25 ± 3.11	33.95 ± 5.38	31.70 ± 2.36
Recurrence	18.48 ± 3.28	27.07 ± 4.71	31.64 ± 4.049	24.85 ± 3.33
*t*	5.81	6.49	2.16	8.70
*P*	.04	.06	.03	.03

#### 3.3.2. Correlation analysis of CRF with anxiety, depression, sleep quality, and social support in elderly NSCLC patients

Pearson correlation analysis was used to assess the relationship between anxiety, depression, sleep quality, social support, and CFS scores at T1-T4 time points. CFS scores at all time points were positively correlated with anxiety, depression, and poor sleep quality, while negatively correlated with social support. Specific results are detailed in Table 5.

**Table 5:** Correlation analysis between cancer-related fatigue and scores of anxiety, depression, insomnia, and social support (n = 108).

Correlation factor	T1 – cancer-related fatigue	T2 – cancer-related fatigue	T3 – cancer-related fatigue	T4 – cancer-related fatigue
T1				
Anxiety	0.549**			
Depressed	0.510**			
Insomnia	0.453**			
Social support	−0.657**			
T2				
Anxiety		0.232*		
Depressed		0.406**		
Insomnia		0.393**		
Social support		−0.579**		
T3				
Anxiety			0.208*	
Depressed			0.304**	
Insomnia			0.403**	
Social support			−0.424**	
T4				
Anxiety				0.537**
Depressed				0.371**
Insomnia				0.586**
Social support				−0.461**

* *P* < .05.

** *P* < .01.

### 3.4. Growth analysis of CRF trajectory subtypes in elderly NSCLC patients

Utilizing Mplus 8.3, an analysis was conducted on the developmental trajectory of CRF in elderly NSCLC patients by fitting CFS scores measured 4 times. Five latent classes of trajectory were designed, with model fit indices such as LL, AIC, BIC, and aBIC decreasing as the number of classes increased, with Entropy consistently above 0.8, indicating better model fit with more latent classes. Despite the bootstrapped likelihood ratio test consistently indicating significance (*P* < .001), the Lo-Mendell-Rubin results were not statistically significant at 4 classes (*P* = .146). Specific results are detailed in Table 6. Thus, this study selected a 3-class latent model.

**Table 6:** Model fitting effect of cancer-related fatigue scores in elderly non-small cell lung cancer patients undergoing chemotherapy.

Category	LL	AIC	BIC	aBIC	Entropy	LMR	BLRT	Scale
1c	−1756.79	3525.58	3541.68	3522.72	–	–	–	–
2c	−1658.87	3335.74	3359.88	3331.44	0.944	<0.001	<0.001	20/88
3c	−1607.59	3239.18	3271.37	3233.45	0.982	0.003	<0.001	53/35/20
4c	−1556.46	3142.92	3183.15	3135.75	0.989	0.146	<0.001	20/35/35/18
5c	−1483.78	2997.58	3037.80	2990.40	0.995	0.151	<0.001	18/6/24/36/24

aBIC = sample size-adjusted BIC, AIC = Akaike information criterion, BIC = Bayesian information criterion, BLRT = bootstrapped likelihood ratio test, c = category, LL = loglikelihood, LMR = Lo-Mendell-Rubin.

**Figure 2.**
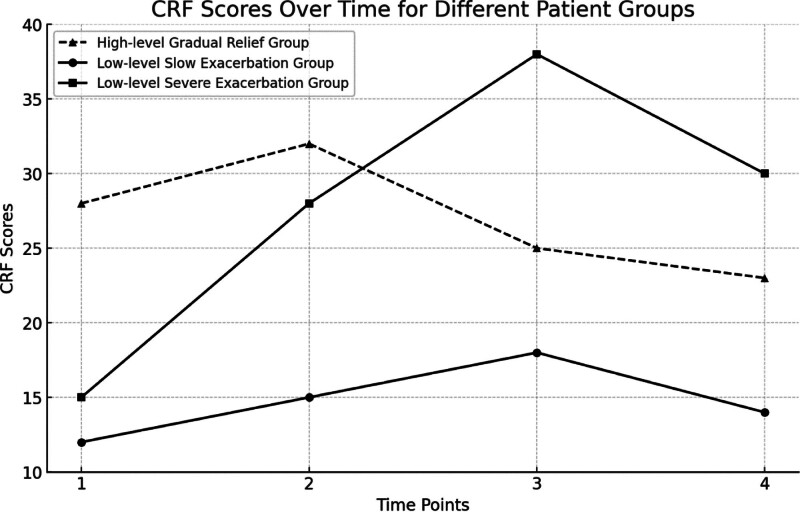
“Low-level slow progression group” comprised 20 individuals (18.5%), “high-level gradual relief group” included 35 individuals (32.4%), “low-level severe aggravation group” consisted of 53 individuals (49.1%).

Group 1 comprised 20 individuals (18.5%), with an intercept of 13.045, indicating an initial average CFS score of 13.045 and a slope of 0.702 (*P* < .05), indicating mild CRF that slowly worsens, termed “low-level slow progression group” in this study. Group 2 included 35 individuals (32.4%), with an intercept of 33.216, indicating an initial average CFS score of 33.216 and a slope of −1.913 (*P* < .05), indicating severe CRF that slowly improves, termed “high-level gradual relief group.” Group 3 consisted of 53 individuals (49.1%), with an intercept of 15.019, indicating an initial average CFS score of 15.019 and a slope of 5.887 (*P* < .01), indicating mild CRF that significantly worsens during treatment, termed “low-level severe aggravation group” in this study. The specific results are shown in Table 7 and Figure 2.

**Table 7:** LCGM estimates for the 3 categories.

Category	Coefficient	
	Intercept (α)	Slope (β)
Low-level slow progression group	13.045**	0.702*
High-level gradual relief group	33.216*	−1.913*
Low-level severe aggravation group	15.019*	5.887**

LGCM = latent growth curve models.

* *P* < .05.

** *P* < .01.

### 3.5. Analysis of factors influencing CRF trajectories in elderly NSCLC patients

#### 3.5.1. Univariate analysis of CRF trajectories in elderly NSCLC patients

Using R × C contingency table analysis, the study’s results indicated that the distribution of disease stages among patients with different CRF trajectories showed statistical significance (*P* < .001). In the low-level slow progression group, high-level gradual relief group, and low-level severe aggravation group, there were 15, 19, and 7 patients in stages I/II, respectively, and 5, 16, and 46 patients in stages III/IV, respectively. The treatment regimen also proved to be a predictive factor. The results showed that patients undergoing radiotherapy and chemotherapy, or surgery combined with radiotherapy and chemotherapy, were more likely to be in the low-level severe aggravation group. See Table 8 for details.

**Table 8:** Univariate analysis of cancer-related fatigue trajectory in elderly non-small cell lung cancer patients (n = 108, %).

Item	Low-level slow progression group	High-level gradual relief group	Low-level severe aggravation group	χ^2^	*P*
Gender					
Male	15 (13.9%)	19 (17.6%)	26 (24.1%)	3.992	.14
Female	5 (4.6%)	16 (14.8%)	27 (25.0%)		
Age					
60 ≤ age < 70	13 (12.0%)	24 (22.2%)	25 (23.1%)	5.351	.25
70 ≤ age < 80	6 (5.6%)	8 (7.4%)	24 (22.3%)		
≥80 yr	1 (0.9%)	3 (2.8%)	4 (3.7%)		
BMI					
BMI < 18.5	4 (3.7%)	15 (13.9%)	20 (18.5%)	7.681	.10
18.5 ≤ BMI < 24	1 (0.9%)	7 (6.5%)	7 (6.5%)		
BMI ≥ 24	15 (13.9%)	13 (12.0%)	26 (24.1%)		
Marital status					
Married	19 (17.5%)	29 (26.9%)	42 (38.9%)	2.603	.27
Unmarried/divorced/widowed	1 (0.9%)	6 (5.6%)	11 (10.2%)		
Monthly family income					
< 3000	6 (5.5%)	14 (13.0%)	13 (12.0%)	5.879	.21
3000 ≤ income < 5000	12 (11.1%)	12 (11.1%)	30 (27.8%)		
≥ 5000	2 (1.9%)	9 (8.3%)	10 (9.3%)		
Insurance status					
Insured	20 (18.5%)	34 (32.5%)	51 (47.2%)	0.767	.68
Self-paying	0 (0.0%)	1 (.9%)	2 (1.8%)		
Disease type					
Squamous cell carcinoma	3 (2.8%)	8 (7.4%)	22 (20.4%)	10.011	.12
Adenocarcinoma	13 (12.0%)	20 (18.5%)	19 (17.6%)		
Large cell carcinoma	1 (0.9%)	4 (3.7%)	3 (2.8%)		
Other	3 (2.8%)	3 (2.8%)	9 (8.3%)		
Disease stage					
Stage I/II	15 (13.9%)	9 (8.3%)	17 (15.7%)	14.659	.00**
Stage III/IV	5 (4.6%)	26 (24.1%)	36 (34.0%)		
Treatment regimen					
Chemotherapy	17 (15.7%)	30 (27.8%)	28 (25.9%)	14.302	.01**
Chemoradiotherapy	2 (1.9%)	3 (2.8%)	20 (18.5%)		
Surgery + chemoradiotherapy	1 (0.9%)	2 (1.9%)	5 (4.6%)		
Recurrence status					
First occurrence	13 (12.0%)	30 (27.8%)	46 (42.6%)	5.147	.07
Recurrence	7 (6.5%)	5 (4.6%)	7 (6.5%)		

BMI = body mass index.

* *P* < .05.

** *P* < .01.

#### 3.5.2. Analysis of the impact of anxiety, depression, sleep quality, and social support on CRF trajectories in elderly NSCLC patients

Among the scores for anxiety, depression, sleep quality, and social support, only sleep quality at T1 showed no correlation with CRF trajectories. At other time points, all 4 indicators were good predictors of patients’ CRF. The study found that patients in the high-level gradual relief group (C2) had higher levels of anxiety and depression and poorer sleep quality compared to the other groups. See Table 9 for details.

**Table 9:** Differences in anxiety, depression, insomnia and social support among elderly non-small cell lung cancer patients in different trajectories (n = 108).

Correlation factor	Time point	Group	Size	Means	Standard	95% confidence interval		*F*	*P*
						Upper limit	Lower limit		
Anxiety	T1	C1	20	8.15	1.93	6.93	9.07	26.96	.00
		C2	35	13.13	1.92	12.07	14.20		
		C3	53	10.27	1.91	9.21	11.32		
	T2	C1	22	9.27	1.94	8.19	10.34	32.11	.000
		C2	32	15.20	2.08	14.05	16.35		
		C3	54	11.73	2.09	10.58	12.89		
	T3	C1	19	8.20	2.54	6.79	9.61	16.58	.001
		C2	37	13.33	2.35	12.03	14.63		
		C3	52	10.47	2.45	9.11	11.82		
	T4	C1	22	7.47	2.77	5.93	9.00	12.93	.000
		C2	33	12.33	2.53	10.93	13.73		
		C3	53	9.40	2.61	7.95	10.84		
Depression	T1	C1	20	9.47	3.02	7.79	11.14	11.04	.000
		C2	35	14.33	2.74	12.81	15.85		
		C3	53	11.40	2.80	9.85	12.95		
	T2	C1	22	10.00	3.38	8.13	11.87	4.03	.025
		C2	32	13.47	3.39	11.59	15.34		
		C3	54	11.92	3.29	10.10	13.74		
	T3	C1	19	9.62	3.27	7.81	11.43	3.27	.048
		C2	37	12.79	3.34	10.94	14.64		
		C3	52	11.31	3.58	9.32	13.29		
	T4	C1	22	8.45	2.80	6.90	10.00	4.5	.017
		C2	33	11.55	2.86	9.97	13.14		
		C3	53	10.10	2.84	8.53	11.67		
Insomnia	T1	C1	20	4.60	2.99	2.94	6.26	3.21	.051
		C2	35	7.40	3.11	5.68	9.12		
		C3	53	6.33	3.06	4.64	8.03		
	T2	C1	22	6.08	3.11	4.36	7.80	3.25	.049
		C2	32	9.02	3.24	7.23	10.81		
		C3	54	7.97	3.26	6.16	9.77		
	T3	C1	19	5.48	2.71	3.98	6.98	3.54	.038
		C2	37	8.07	2.73	6.56	9.58		
		C3	52	6.61	2.57	5.19	8.03		
	T4	C1	22	4.69	2.48	3.32	6.06	4.19	.022
		C2	33	7.30	2.61	5.86	8.74		
		C3	53	5.82	2.35	4.52	7.12		
Social support	T1	C1	20	46.40	6.66	42.71	50.09	51.27	.000
		C2	35	21.80	6.67	29.51	36.89		
		C3	53	33.20	6.67	18.11	25.49		
	T2	C1	22	48.07	6.85	44.27	51.86	45.24	.000
		C2	32	24.33	6.86	20.53	28.13		
		C3	54	34.53	6.85	30.74	38.33		
	T3	C1	19	50.07	8.44	45.39	54.74	29.63	.000
		C2	37	26.33	8.86	21.43	31.24		
		C3	52	36.53	8.10	32.05	41.02		
	T4	C1	22	51.40	7.21	47.41	55.39	37.94	.000
		C2	33	28.33	7.34	24.27	32.40		
		C3	53	38.53	7.26	34.51	42.55		

C1: low-level slow progression group; C2: high-level gradual relief group; C3: low-level severe aggravation group.

#### 3.5.3. Multinomial logistic regression analysis of CRF trajectories in elderly NSCLC patients

Following univariate analysis, the study identified disease stage, treatment regimen, levels of anxiety and depression, sleep quality, and social support as statistically significant independent variables that influence the CRF trajectories in elderly NSCLC patients. Using the three CRF trajectories as dependent variables and the low-level slow progression group as the reference, these independent variables were subjected to a multinomial logistic regression analysis to determine the predictive indicators for CRF trajectories in elderly NSCLC patients. The coding for the independent variables is detailed in Table 10, and continuous variables were entered as their original values. See Table 11 for detailed results.

**Table 10:** Assignment of independent variables and dependent variables in regression analysis.

Variable	Assignment
Trajectory	Low-level slow progression group = 1, high-level gradual relief group = 2, low-level severe aggravation group = 3
Disease stage	Stage I/II = 1, Stage III/IV = 2
Treatment regimen	Chemotherapy = 1, chemoradiotherapy = 2, surgery + chemoradiotherapy = 3
Anxiety	Enter the original value
Depressed	Enter the original value
Insomnia	Enter the original value
Social support	Enter the original value

**Table 11:** Parameter estimation results of unordered multi-classification logistic regression analysis (n = 108).

Dependent variable	Independent variable	B	SE	Wald χ^2^	OR	95% confidence interval		*P*
						Upper limit	Lower limit	
C2	Disease stage							
	Stage III/IV	2.787	1.106	6.348	0.062	0.007	0.539	.012
	Anxiety T1	2.891	1.099	6.917	8.006	2.089	15.235	.009
	Insomnia T2	0.915	0.163	21.560	2.541	1.997	3.718	.001
	Social support T4	−0.237	0.062	13.818	0.972	0.892	1.125	.031
C3	Disease stage							
	Stage III/IV	3.027	1.144	7.003	0.048	0.005	0.456	.008
	Treatment regimen							
	Chemoradiotherapy	3.253	1.258	6.690	0.039	0.003	0.455	.010
	Anxiety T1	3.011	1.519	7.956	9.506	4.197	16.515	.018
	Depressed T1	2.517	1.085	5.376	6.387	1.476	10.959	.020
	Insomnia T2	0.863	0.124	19.462	2.406	1.716	3.014	.023
	Social support T4	−1.237	0.762	15.117	1.871	1.015	2.572	.001

## 4. Discussion

This study conducted a longitudinal tracking of CRF with a total of 4 assessments. Following the determination of the chemotherapy regimen (T1), the first assessment revealed that the vast majority of patients experienced mild to moderate CRF, with an average score of 19.3. This finding may be closely related to the patients’ disease state. Most patients were hospitalized due to symptoms such as coughing and hemoptysis, facing an unfamiliar and uncomfortable ward environment, frequent medical procedures, and the psychological stress of disease diagnosis. Additionally, the tumor itself might trigger fluctuations in cytokine and hormone levels, affecting the endocrine and immune systems’ functions and further exacerbating the severity of CRF. Consequently, patients exhibited CRF at the early stages of the treatment regimen.^[45]^ After completing the first round of chemotherapy (T2), patients generally experienced varying degrees of increased fatigue, with an average score of 28.4, primarily affecting physical and cognitive aspects. Chemotherapy often causes severe gastrointestinal side effects such as nausea, vomiting, and diarrhea, which reduce patients’ appetites, leading to malnutrition and physical exhaustion. Additionally, chemotherapy-induced myelosuppression further disrupts the immune system, lowering blood cell counts, which is a significant factor contributing to the patients’ sense of fatigue.^[46]^ Therefore, this reminds the care team to pay special attention to fall prevention when caring for elderly patients undergoing chemotherapy. They should actively provide health education, guiding patients to adopt a light diet and increase fluid intake during chemotherapy to help eliminate toxins from the body and reduce the drug’s irritation to the gastrointestinal mucosa.

After the first round of chemotherapy, patients’ emotional fatigue levels significantly increased. This phenomenon may stem from the initial chemotherapy failing to noticeably alleviate pathological symptoms, combined with the negative experience of the first chemotherapy increasing patients’ anxiety. Additionally, patients’ lack of understanding about the disease and chemotherapy side effects might lead to issues such as disrupted self-identity and anticipatory grief, further increasing psychological stress. Therefore, caregivers should emphasize psychological care, providing timely positive guidance and expanding patients’ knowledge related to the disease.^[47–49]^ The neurotoxicity of chemotherapy drugs can lead to peripheral nerve symptoms such as numbness, tingling pain, and muscle cramps in the extremities,^[50]^ These symptoms not only significantly increase patient discomfort but may also affect the central nervous system, potentially causing chemotherapy-related cognitive impairment.^[51]^ As chemotherapy progresses, this study observed that patients’ cognitive fatigue gradually increases. Therefore, caregivers should pay special attention to this issue during the care process and use methods such as narrative care, exercise guidance, and visual training to alleviate patients’ cognitive fatigue, thereby improving their overall well-being.^[52–54]^

After completing the fourth round of chemotherapy (T3), both physical and cognitive fatigue reached their highest measured levels in this study, with a score of 32.06, while emotional fatigue significantly decreased. This change might be related to the visible effects of chemotherapy, as patients felt optimistic about the treatment results, gained a deeper understanding of their condition, and gradually adapted to the chemotherapy process, leading to a notable relief in emotional fatigue. At this stage, emphasis should be placed on patients’ dietary and exercise care, recommending flexible dietary arrangements and moderate physical activity.

The fourth survey of this study was conducted when patients were discharged (T4). Although overall fatigue levels decreased as treatment ended, the average score was still 26.1, higher than at the initiation of the treatment plan. At this point, both physical and emotional fatigue had improved, but cognitive fatigue remained at a high level. The effects of chemotherapy drugs on the central nervous system may persist for a long time, resulting in slower relief of cognitive impairment, with some patients potentially being affected for an extended period.^[55]^ Therefore, caregivers should guide patients in both physical and cognitive exercises, and employ cognitive behavioral therapy. Cognitive therapy primarily uses education, cognitive restructuring, and role-playing to effectively correct cognitive biases, eliminate negative emotions and inappropriate behaviors, improve sleep quality, and reduce psychological stress. Behavioral therapy focuses on similar cognitive training. This approach mainly encourages patients to engage in relaxation training, role-playing, group activities, and behavior interruption. By learning and adapting to new behavior patterns, it aims to reduce the impact of cognitive impairments on daily life quality.^[51]^ At the same time, adjusting diet structure and lifestyle, and guiding family members on how to provide psychological support to the patient are crucial. As chemotherapy concludes, most patients’ chemotherapy-related cognitive impairment will gradually diminish. Overall, this study shows that the CRF status of patients undergoing radiotherapy continuously changes, and these changes are statistically significant. Therefore, caregivers should promptly assess patients’ CRF and implement targeted care measures based on the level of fatigue.

Previous studies indicate that age is a factor influencing CRF, but findings are heterogeneous. For instance, research on leukemia patients suggests that as age increases, tolerance to chemotherapy decreases, resulting in higher levels of fatigue.^[56]^ Conversely, other studies suggest that younger patients may experience severe fatigue more readily,^[57,58]^ possibly due to their higher baseline energy levels; young patients often feel a significant energy deficit after chemotherapy, compounded by greater life pressures, potentially intensifying CRF. However, in this study, age did not show a significant impact on CRF. This could be because the study focused on elderly patients whose fatigue thresholds may be higher due to declining physical function, thus not showing statistically significant changes in fatigue perception. Therefore, age may not be an effective assessment indicator for CRF in evaluating elderly NSCLC chemotherapy patients, Additionally, previous research suggests that due to hormonal fluctuations, female patients may experience higher levels of CRF.^[59]^ However, in this study, there was no statistically significant difference in CRF between genders, which may be attributed to decreased ovarian function and hormonal fluctuations in elderly women. BMI also did not emerge as a factor influencing patients’ CRF, consistent with prior research.^[34]^ The study further indicated that factors such as BMI, average monthly income, marital status, and insurance coverage did not significantly impact CRF. This could be related to the majority of patients receiving treatment in tertiary hospitals with broad insurance coverage, resulting in lower medical pressures. Additionally, while tumor pathology itself was not a decisive factor for CRF, disease staging showed a significant influence. Due to limited understanding of tumor pathology, patients may have insufficient knowledge, whereas disease staging information is easier to comprehend, leading to greater stress and stronger pathological responses among Stage III/IV patients, resulting in more intense initial fatigue levels. However, as chemotherapy progresses, the fatigue levels of Stage I/II patients may increase due to the adverse effects of chemotherapy, potentially surpassing those of Stage III/IV patients later in the treatment. This suggests that healthcare providers need to conduct dynamic assessments and timely interventions for CRF in Stage I/II patients to prevent its deterioration and impact on quality of life. Additionally, recurrent patients experienced less fatigue compared to first-time patients, possibly due to their prior medical experience and psychological preparedness. Therefore, the results of this study emphasize that healthcare providers should pay special attention to CRF in patients undergoing chemo radiotherapy, those in advanced stages of the disease, and newly diagnosed patients, and implement targeted care measures.

In this study, it was observed that patients’ CRF levels were positively correlated with anxiety, depression, and sleep quality, and negatively correlated with levels of social support, consistent with previous research.^[60]^ A similar study on breast cancer patients also found that those with anxiety and depression symptoms were more likely to develop severe fatigue.^[61]^ Patients with severe negative emotions typically have lower expectations for life, which can lead to poor medical compliance and exacerbate fatigue symptoms. Therefore, caregivers should focus on helping patients enhance their positive emotions.^[62]^ Research indicates that positive emotions not only foster creative actions, ideas, and social connections,^[63]^ but also help build personal resources across material, intellectual, social, and psychological domains, thereby alleviating CRF.^[34]^ Sleep disorders are another significant factor affecting CRF.^[57]^ Good sleep quality is key to preventing and mitigating CRF,^[64]^ This finding highlights the need for healthcare providers to pay more attention to patients with poor sleep quality, promptly assess their CRF levels, and take measures to improve sleep quality.^[65]^ These measures can include establishing regular sleep habits, providing a comfortable sleep environment, or using nonpharmacological treatments such as cognitive behavioral therapy to address sleep disorders.^[66]^ The role of social support is also crucial. Research shows that a strong social support network can effectively reduce patients’ psychological stress, enhance their coping abilities, and improve their quality of life.^[67]^ Therefore, enhancing patients’ social support is an important strategy for reducing CRF. Caregivers can increase patients’ perceived social support by encouraging participation in support groups, promoting communication with family and friends, or connecting patients with social service resources.

Based on the time-experience symptom model, CRF in elderly NSCLC patients demonstrates dynamic changes. This study employed latent growth modeling to analyze patients’ CRF and successfully identified 3 latent subgroups, consistent with CRF development trajectories observed in previous lung cancer studies.^[35]^ These 3 categories are labeled as the “high-level gradual relief group,” the “low-level slow increase group,” and the “low-level sharp increase group.” Specifically, the “high-level gradual relief group” comprises 32% of patients who initially exhibit moderate to severe CRF, with significant fatigue at T1, peaking at T2, and then gradually decreasing with treatment, but still maintaining moderate fatigue levels, similar to previous research.^[57,68]^ In contrast, the “Low-level Slow Increase group” accounts for 19% of patients. These individuals start with relatively low levels of CRF, and although fatigue levels increase gradually during chemotherapy, they generally remain at lower levels overall, consistent with prior research.^[61]^ Of particular note is the “Low-level Sharp Increase group,” comprising the largest proportion at 49%. These patients are highly sensitive to chemotherapy’s adverse effects, experiencing continuous escalation of CRF throughout treatment to severe fatigue levels. Their fatigue only slightly alleviates by discharge, yet they still remain in a state of severe fatigue. Therefore, caregivers should early assess patients’ fatigue trajectories based on their demographics, anxiety, depression, and other indicators, and implement targeted measures to optimize treatment and care plans. Such strategies not only enhance patients’ quality of life but also contribute to improving their overall treatment response.

Through multi-class logistic regression analysis incorporating patients’ demographic data, disease-related factors, psychological states, sleep quality, and social support levels, this study identified several key predictive factors. These include disease staging, levels of anxiety and depression, sleep quality, and social support levels, which were proven effective indicators for predicting the development trajectories of CRF during chemotherapy in elderly NSCLC patients. The study found that general demographic statistics alone are insufficient for predicting CRF, possibly due to the elderly focus of the study population. In contrast, disease staging emerged as a robust predictor, with late-stage patients more likely to follow trajectories such as the “low-level sharp increase group” or the “high-level gradual relief group.” These patients, due to the severity of their illness, poor experiences, greater emotional fluctuations, higher prechemotherapy CRF levels, and increased treatment-related anxieties, exhibit faster exacerbation of fatigue. However, early-stage (I/II) patients should not be overlooked; although their initial fatigue levels are lower, some may still progress to the “Low-level Sharp Increase group.” Therefore, healthcare providers should prioritize late-stage patients and conduct dynamic assessments of fatigue levels in early-stage patients to promptly identify and intervene. Previous studies indicate that anxiety and depression are independent risk factors for CRF in patients.^[69]^ This study found that in elderly NSCLC chemotherapy patients, levels of anxiety and depression are independent predictors. The more severe the negative emotions, the more likely patients are to eventually develop either severe escalation in low-level fatigue or gradual relief in high-level fatigue.^[70]^ Prior research also indicates that positive emotions play a mediating role in the relationship between psychological resilience and CRF.^[34]^ Therefore, for patients with higher psychological resilience, healthcare providers should provide psychological counseling and educational support, maintain timely communication, and promptly identify CRF symptoms to prevent worsening fatigue levels. Sleep quality is also a predictor of whether patients will develop severe escalation in low-level fatigue or gradual relief in high-level fatigue. Previous studies have shown that patient sleep quality helps healthcare providers predict the trajectory of patient fatigue,^[71]^ Hence, attention should be paid to patient sleep quality, and active interventions should be taken for patients with poor sleep quality to avoid severe worsening of fatigue levels. Social support is also a predictor of severe escalation in low-level fatigue, consistent with previous research,^[68]^ Higher social support slows down the growth of patient fatigue levels. Therefore, efforts should be made to help patients build support networks, enhancing their sense of support and happiness.

This study adopted a longitudinal design to track the progression of CRF across 4 chemotherapy stages in elderly patients with NSCLC. Compared to cross-sectional methods, this approach revealed dynamic fatigue patterns over time and identified key predictive factors such as disease stage, anxiety, depression, sleep quality, and social support. These variables served as early warning indicators, enabling care teams to identify high-risk patients and implement timely, individualized interventions. By integrating psychosocial and fatigue assessments into routine care, healthcare professionals improved treatment adherence and reduced symptom burden. Moreover, the trajectory-based prediction model enhanced the ability of clinicians to anticipate patient needs and optimize resource allocation throughout the treatment process. Collectively, these findings reinforced the need to address CRF as a multifactorial condition shaped by physiological, emotional, and social factors, calling for coordinated, multidisciplinary care strategies. The study also offered valuable guidance for institutional policy-making, nursing education, and caregiver training, particularly within the context of geriatric oncology.

Although this study has strived to ensure scientific rigor in its design, it undeniably has several limitations that need improvement. (1) Due to constraints such as time and personnel, the study was conducted in only one tertiary hospital with a relatively small sample size, which may limit the generalizability of the results. Additionally, CRF is a dynamic symptom that persists over a long period, and patients may continue to experience fatigue long after discharge. The follow-up period in this study was short, focusing only on the development trajectory of CRF during hospitalization, which does not provide a comprehensive guide for managing CRF postdischarge. Future studies should consider conducting multicenter investigations with larger sample sizes and longer follow-up periods to better delineate the development trajectory of CRF. (2) According to previous research, the factors associated with CRF are complex and multifaceted, involving aspects such as red blood cell count, levels of inflammatory markers, hope levels, nutritional status, supportive care, and psychological resilience. However, due to limitations in study time and sample size, these variables were not included in this study. Future research could expand the sample size and include a more comprehensive set of relevant factors to improve the study. (3) While this study has characterized and analyzed the development trajectory and predictive factors of CRF in elderly NSCLC chemotherapy patients, it did not conduct specific interventional research to indicate how to manage patient fatigue. Further research is needed to explore specific nursing interventions for managing patient fatigue to guide clinical practice.

## 5. Conclusion

In elderly NSCLC chemotherapy patients, CRF exhibits nonlinear dynamic changes. As chemotherapy progresses, overall CRF levels gradually increase, decrease towards the end of treatment, but remain elevated compared to pretreatment levels. CRF is prevalent among elderly NSCLC chemotherapy patients and worsens with chemotherapy. Multiple factors influence CRF, primarily disease-related characteristics such as disease stage, treatment regimen, and recurrence, as well as psychological status, sleep quality, and social support. CRF manifests in several potential categories, identifying 3 distinct trajectories: low-level slow exacerbation group, high-level gradual alleviation group, and low-level severe exacerbation group. Disease stage, levels of anxiety and depression, sleep quality, and social support have been identified as predictive factors for CRF trajectories in elderly NSCLC chemotherapy patients.

## Author contributions

**Conceptualization:** Xiuhong Wei.

**Data curation:** Chen Liu, Hao Wang, Taotao Xu, Han Wang, Xiuhong Wei.

**Formal analysis:** Chen Liu, Hao Wang, Taotao Xu.

**Funding acquisition:** Hao Wang.

**Investigation:** Chen Liu, Hao Wang.

**Methodology:** Chen Liu, Hao Wang, Xiuhong Wei.

**Project administration:** Taotao Xu.

**Resources:** Hao Wang, Taotao Xu, Han Wang.

**Software:** Hao Wang, Han Wang, Yan Zhai.

**Supervision:** Han Wang.

**Validation:** Jiaxuan Wen, Xiuhong Wei, Yan Zhai.

**Visualization:** Jiaxuan Wen, Xiuhong Wei, Yan Zhai.

**Writing – original draft:** Jiaxuan Wen, Yan Zhai.

**Writing – review & editing:** Jiaxuan Wen, Yan Zhai.
